# Efficacy and safety of DFN-11 (sumatriptan injection, 3 mg) in adults with episodic migraine: a multicenter, randomized, double-blind, placebo-controlled study

**DOI:** 10.1186/s10194-018-0881-z

**Published:** 2018-08-15

**Authors:** Stephen Landy, Sagar Munjal, Elimor Brand-Schieber, Alan M. Rapoport

**Affiliations:** 1 Baptist Medical Group Headache Clinic, University of Tennessee Medical School, 6029 Walnut Grove, Suite 210, Memphis, TN 38120 USA; 2 Promius Pharma, a subsidiary of Dr Reddy’s Laboratories, 107 College Road East, Princeton, NJ 08540 USA; 3 The David Geffen School of Medicine at UCLA, 4255 Jefferson Avenue, Suite 27, Woodside, CA 94062 USA

**Keywords:** Episodic migraine, Migraine treatment, Subcutaneous sumatriptan, Sumatriptan autoinjector

## Abstract

**Background:**

In a previous randomized, double-blind, proof-of-concept study in rapidly escalating migraine, a 3 mg dose of subcutaneous sumatriptan (DFN-11) was associated with fewer and shorter triptan sensations than a 6 mg dose. The primary objective of the study was to assess the efficacy and safety of acute treatment with DFN-11 compared with placebo in episodic migraine.

**Methods:**

This was a multicenter, randomized, double-blind, placebo-controlled efficacy and safety study of DFN-11 in the acute treatment of adults with episodic migraine (study RESTOR). The primary endpoint was the proportion of subjects taking DFN-11 who were pain free at 2 h postdose in the double-blind period compared with placebo. Secondary endpoints included earlier postdose timepoints, assessments of pain relief and subjects’ freedom from their most bothersome symptom (MBS) (among nausea, photophobia, and phonophobia). Safety and tolerability were assessed.

**Results:**

A total of 392 subjects was screened, 268 (68.4%) were randomized, and 234 (87.3% of those randomized) completed the double-blind treatment period. The proportion of subjects who were pain free at 2 h postdose was significantly greater in the DFN-11 group than in the placebo group (51.0% vs 30.8%, *P*  =  0.0023). Compared with placebo, significantly higher proportions of subjects treated with DFN-11 were also pain free at 30, 60, and 90 min postdose (*P*  ≤  0.0195). DFN-11 was significantly superior to placebo for pain relief at 60 min, 90 min, and 2 h postdose (*P* ≤ 0.0179). At 2 h postdose, DFN-11 was also significantly superior to placebo for freedom from photophobia (*P*  =  0.0056) and phonophobia (*P*  =  0.0167). Overall, 33.3% (37/111) who received DFN-11 and 13.4% (16/119) who received placebo experienced at least 1 treatment-emergent adverse event (TEAE), the most common of which were injection site swelling (7.2% vs 0.8%) and pain (7.2% vs 5.9%). Chest discomfort was about half as common in the DFN-11 treatment group as it was in the placebo group (0.9% vs 1.7%).

**Conclusions:**

This study met its primary endpoint, pain freedom at 2 h postdose, with DFN-11 significantly better than placebo, and the incidence of TEAEs and triptan sensations with DFN-11 was low. The 3 mg dose of sumatriptan in DFN-11 appears to be an effective alternative to a 6 mg SC dose of sumatriptan, with good safety and tolerability. (clinicaltrials.gov: NCT02569853; registered 07 October 2015).

**Electronic supplementary material:**

The online version of this article (10.1186/s10194-018-0881-z) contains supplementary material, which is available to authorized users.

## Background

Migraine is a chronic neurologic disorder characterized by episodic attacks of head pain and associated symptoms, such as photophobia, phonophobia, and gastrointestinal disturbances; attacks are often accompanied by cutaneous allodynia and may be preceded by an aura and/or premonitory symptoms [1]. The International Headache Society recognizes 2 main subtypes: episodic migraine, with fewer than 15 headache days per month, and chronic migraine, in which headache is present on 15 or more days per month [1]. In the United States, nearly 38 million adults have migraine [2, 3], with women about 3 times more likely to be affected than men (18% vs 6%) [4]. For many patients, the burden of migraine includes negative effects on performance and attendance at work, school, family, and leisure activities. Migraineurs have an increased likelihood of unemployment, lack of advancement at work, and occupational disability, as well as elevated risk of developing comorbid conditions (eg, depression, anxiety and other pain disorders) [5–7].

The acute treatment of migraine may involve simple analgesics, combination over-the-counter medications, nonsteroidal anti-inflammatory drugs (NSAIDs), and migraine-specific medications (eg, triptans and ergot alkaloids) [8–13]. Triptans are often considered the best choice for first-line therapy [8, 9, 14]. The most rapidly effective treatment in the class is 6 mg subcutaneous (SC) sumatriptan [15, 16], which reaches peak plasma concentration (t_max_) in 12 min, has an onset of action of 10 min, and relieves migraine pain in 82% of patients at 2 h postdose [17]. Despite its excellent efficacy profile, fewer than 10% of migraineurs who might benefit from SC sumatriptan use it to treat their condition [18]. This may be because most (59%) SC sumatriptan-treated patients have injection site reactions, and many (42%) experience triptan sensations (eg, tingling, warm/hot, tightness/pressure) that appear to be dose-related [19]. Concerns about drug-related adverse events (AEs) have caused two thirds of migraine patients to delay or avoid treating an attack [18].

DFN-11 (Zembrace® SymTouch®, Promius Pharma, Princeton, NJ) is a low-dose (3 mg) sumatriptan SC injection supplied as a single-dose, ready-to-use, disposable autoinjector [20], which distinguishes it from the 6 mg SC dose of sumatriptan (Imitrex®, GlaxoSmithKline, Research Triangle Park, NC). DFN-11 has less sumatriptan per dose (3 mg vs 6 mg) and is therefore a more dilute formulation (3 mg/0.5 mL vs 6 mg/0.5 mL) [17, 21]. It is stable at controlled room temperature storage and has a 2-year shelf life. In an earlier pilot study in adults with rapidly escalating migraine attacks, DFN-11 was shown to be as effective as a 6 mg SC dose of sumatriptan, and was associated with improved tolerability [22]. Specifically, subjects who received DFN-11 had effective relief of migraine pain and associated symptoms, a lower incidence of triptan sensations, and no chest pain [22]. The objective of the present study was to assess the efficacy, tolerability, and safety of DFN-11 in the acute treatment of episodic migraine attacks.

## Methods

### Ethics

This randomized, double-blind, placebo-controlled study, with an open-label extension, evaluated the efficacy, tolerability, and safety of DFN-11 in adults with episodic migraine (study RESTOR). It was conducted at 17 study centers in the United States. The protocol was approved by the institutional review boards at each study site, and the study was conducted in compliance with good clinical practice and in accordance with the ethical principles set forth in the Declaration of Helsinki. Before any study-specific procedures were initiated, investigators explained the nature of the study to the subjects, and subjects provided informed consent. Details about the trial are available online at ClinicalTrials.gov (Identifier: NCT02569853).

### Subjects

Subjects included males and females aged 18 to 65 years with a history of episodic migraine with or without aura who experienced 2 to 6 migraine attacks per month for at least the previous 12 months, with no more than 14 headache days per month and a minimum of 48 h of headache-free time between attacks. Individuals with aura lasting longer than 60 min were excluded. Female subjects had to have a negative serum pregnancy test at screening and all subsequent study visits and no plans to become pregnant during the study, and they could not be lactating. Female subjects with male partners and male subjects with female partners had to agree to practice a reliable form of contraception or abstinence during the study.

Subjects were excluded if they had medication overuse headache or any abnormal physiology and/or pathology that would compromise data collection, confound the objectives of the study, be a contraindication for study participation, or not allow the objectives of the study to be met. Complete exclusion criteria are provided in the Additional file 1 (available online).

### Study procedures

This study involved 5 site visits: screening, baseline/randomization, end double-blind/begin open-label, week 4, and end of study visit.

Screened subjects who met all the inclusion and none of the exclusion criteria were instructed by the site staff on the proper administration of study medication and randomized (1:1) to receive DFN-11 or placebo via SC autoinjector in a double-blinded fashion.

In the double-blind period, which lasted 4 weeks, subjects self-administered DFN-11 or placebo via SC autoinjector to treat 1 migraine attack within 1 h of experiencing pain of moderate to severe intensity. Subjects who experienced insufficient relief from the first dose of study medication were permitted to take a second dose of study medication or rescue medication if at least 2 h had elapsed since the first dose, and they had completed electronic diary (eDiary) ratings up to and including the 2-h postdose rating. No more than 2 doses of study medication were allowed in any 24-h period. Rescue medications could include prescription and nonprescription drugs (eg, NSAIDs, other acute migraine medications, vitamins, herbal/dietary supplements). At the conclusion of the double-blind treatment period, subjects were re-assessed for eligibility before for continuing into an 8-week open-label period [23].

During the study, eDiaries were used to record data in real-time and transmit it to a web-based data storage system. Diary assessments included onset and duration of headache, predose severity of pain, associated symptoms (including the most bothersome), functional disability, rescue medication use, and injection site reactions.

### Assessments

#### Efficacy

The primary efficacy endpoint was the proportion of subjects with moderate to severe predose pain who were pain free 2 h after the taking first dose of medication, comparing DFN-11 and placebo. Secondary efficacy variables in the double-blind treatment period included freedom from nausea, photophobia, and phonophobia at 10, 15, 20, 30, 60, 90 min, and 2 h postdose; pain relief at 10, 15, 20, 30, 60, 90 min, and 2 h postdose; pain freedom at 10, 15, 20, 30, 60, and 90 min postdose); MBS absent at 10, 15, 20, 30, 60, 90 min, and 2 h postdose; sustained pain freedom at 24 h (2–24 h) postdose; and use of rescue medication or a second dose of the study medication after 2 h (2–24 h) postdose.

Pain intensity was graded on a 4-point Likert-type [24] scale where 0  =  no pain, 1  =  mild pain, 2  =  moderate pain, and 3  =  severe pain. Pain free was defined in the double-blind period as a reduction in pain intensity from moderate or severe at baseline to none at 2 h postdose. Pain relief was defined as a reduction from moderate or severe baseline pain to mild or none at 2 h postdose. The MBS was defined as the symptom associated with migraine that was the most bothersome from among nausea, photophobia, and phonophobia identified prior to dosing. Freedom from the MBS was defined as the absence of the baseline MBS at 2 h postdose. Sustained pain freedom was defined as pain-free at 2 h postdose with no use of rescue medication or additional study medication and no recurrence of headache pain within 2 to 24 h postdose.

#### Safety

The safety endpoints in this study were the proportion of subjects with treatment-emergent AEs (TEAEs) and serious AEs (SAEs), as well as those with changes in clinical laboratory tests, vital signs, and ECG. Triptan related AE terms included, but were not limited to, tingling/prickling, dizziness/vertigo, warm/hot/burning sensation, cold sensation, feeling of heaviness/pressure/tightness, paresthesia, flushing, and numbness. For injection site reactions, terms included injection site swelling, irritation, erythema, hemorrhage, pain, and bruising.

### Statistics

The randomization list was generated by study personnel who were not involved with study conduct, and the allocation of randomization numbers to study drug kits was performed by a third-party vendor. In the double-blind portion of the study, all subjects and study personnel involved with study conduct were blinded to the drug assignment. For quantitative variables, descriptive statistics include the number of subjects (*n*), mean, standard deviation (SD), median, minimum, and maximum. Categorical variables are summarized using the number (*n*) and percentage (%) of subjects for each category. All data processing, summarization, and analyses were performed using SAS® software, Version 9.2. Adverse events were classified using the Medical Dictionary for Regulatory Activities (MedDRA) dictionary, Version 18.0. All concomitant medications were coded using the World Health Organization Drug Dictionary Enhanced (WHODDE), version Mar2015 further coded against Anatomic Therapeutic Chemical (ATC) classification.

Multiple study populations were analyzed. Subjects who were randomized in the double-blind period were the basis of the subject disposition and baseline summaries. Those who were randomized, received at least 1 dose of double-blind study medication, and recorded at least 1 postdose double-blind efficacy data point were used for the double-blind efficacy analyses.

The analyses comparing proportions were done with 1-sided Fisher’s exact test with an alpha level equal to 0.025 to determine statistical significance. Unless noted otherwise, a last observation carried forward (LOCF) imputation method was applied. Baseline data were not carried forward, and only valid data from postbaseline assessments collected before the 2-h postdose time point were carried forward to impute the next missing assessment up to the 2-h postdose time point. Time points beyond 2 h postdose were not carried forward.

The analysis of safety in the double-blind treatment period was based on data from all randomized subjects who received at least 1 dose of study medication in the double-blind period. The efficacy analyses were based on postdose timepoints data captured in real-time in an eDiary for migraine attacks treated. Safety assessment at baseline was defined as the last assessment before receiving the first dose of study medication in the double-blind period. Change from baseline was defined as the postbaseline value minus the baseline value, and the calculations were based on nonmissing data.

One or more interim analyses were planned for the purpose of evaluating potential early stopping for an efficacy conclusion after a minimum of 166 subjects had completed the double-blind period and primary endpoint data were available for approximately 145 subjects. A conservative alpha spending function was used to preserve most of the type I error for the final analysis in case the trial did not stop for an efficacy conclusion at the interim analysis.

## Results

### Subjects

Sixteen sites in the United States participated and randomized subjects in the study. The first subject was enrolled on 21 September 2015, and the last subject completed the study on 30 May 2017.

**Fig. 1:**
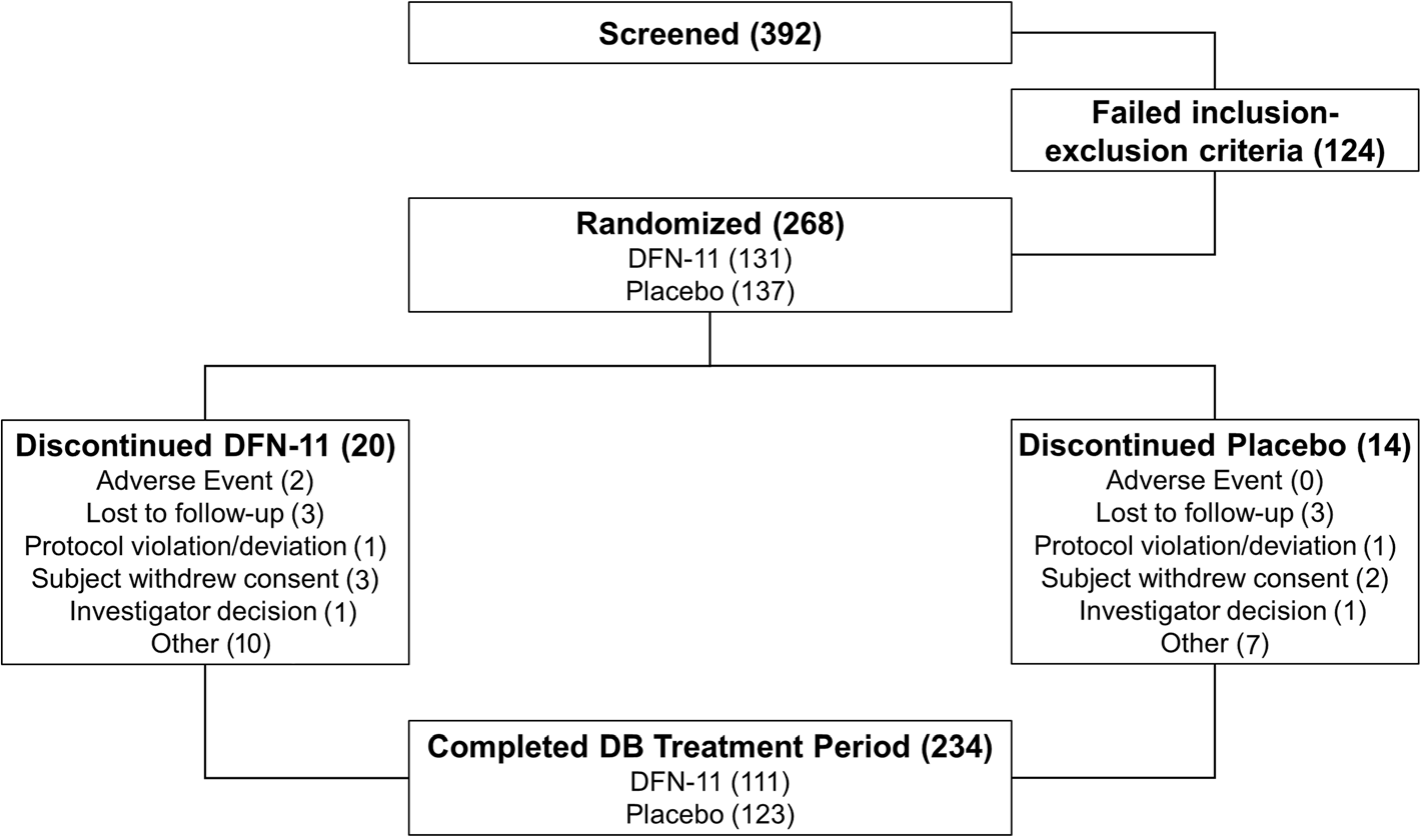
Disposition of subjects

A total of 392 subjects was screened, 268 (68.4%) were randomized, and 234 (87.3% of those randomized) completed the double-blind treatment period (Fig. 1). In all, 34 (12.7%) subjects discontinued from the study. Two (0.7%) subjects discontinued from the study due to AEs with DFN-11.

As Table 1 shows, subjects in the DFN-11 and placebo treatment groups were demographically similar. The mean (SD) age of the study population was 41.0 (12.4) years, with subjects in the DFN-11 group slight older than those assigned to receive placebo (41.9 [12.5] vs 40.2 [12.3]). Mean (SD) weight was nearly identical between those receiving DFN-11 and placebo (84.4 [23.9] kg vs 84.3 [23.6] kg), as was body mass index (30.4 [8.4] kg/m^2^ vs 30.7 [8.8] kg/m^2^). Overall, most subjects were female (85.4%) and white (75.7%), and the DFN-11 and placebo treatment groups had roughly equal proportions of female (84.7% vs 86.1%, respectively) and white subjects (74.8% vs 76.6%, respectively).

**Table 1:** Demographics

	DFN-11 (*n* = 131) *n* (%)	Placebo (*n* = 137) *n* (%)	Overall (*N* = 268) *n* (%)
Age, years^a^	41.9 (12.5)	40.2 (12.3)	41.0 (12.4)
Sex			
Men	20 (15.3)	19 (13.9)	39 (14.6)
Women	111 (84.7)	118 (86.1)	229 (85.4)
Race			
Asian	1 (0.8)	3 (2.2)	4 (1.5)
Black/African-American	29 (22.1)	23 (16.8)	52 (19.4)
Native Hawaiian or Other Pacific Islander	0 (0.0)	2 (1.5)	2 (0.7)
White	98 (74.8)	105 (76.6)	203 (75.7)
Other	3 (2.3)	4 (2.9)	7 (2.6)
Ethnicity			
Not Reported	0 (0.0)	1 (0.7)	1 (0.4)
Hispanic or Latino	10 (7.6)	9 (6.6)	19 (7.1)
Not Hispanic or Latino	121 (92.4)	127 (92.7)	248 (92.5)
Weight, kg^a^	84.4 (23.9)	84.3 (23.6)	84.4 (23.7)
Height, cm^a^	166.7 (8.4)	166.0 (8.7)	166.4 (8.5)
Body Mass Index, kg/m^2a^	30.4 (8.4)	30.7 (8.8)	30.6 (8.6)

^a^Mean (SD)

### Efficacy

**Fig. 2:**
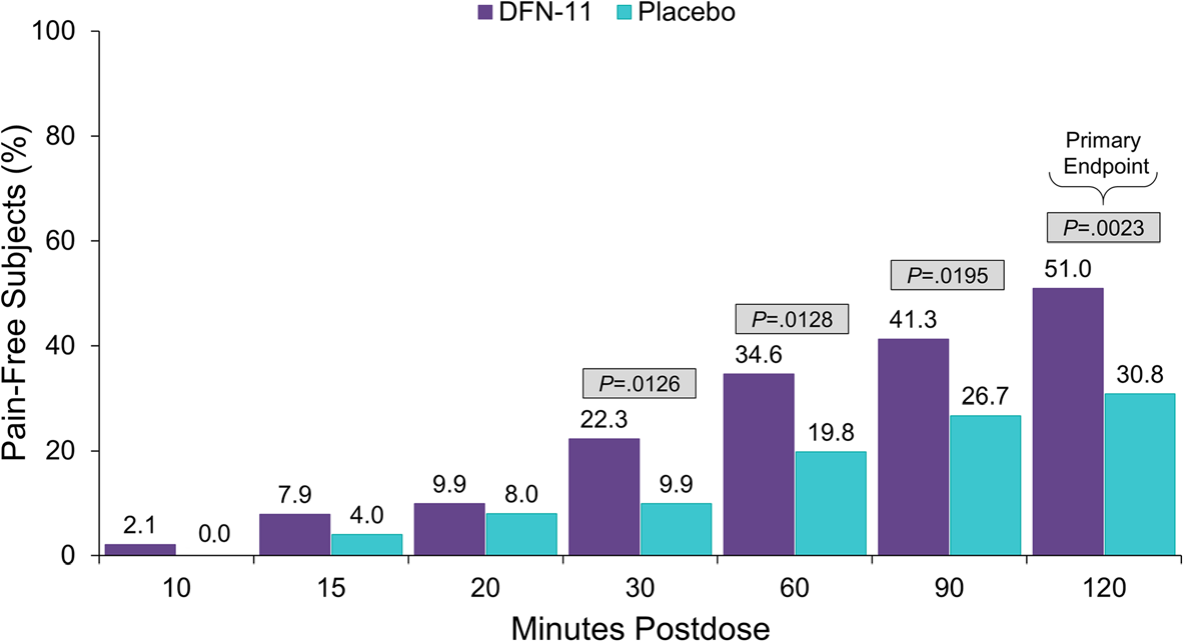
Proportion of subjects with pain freedom through 2 h postdose

The proportion of subjects who were pain free at 2 h postdose — the prespecified primary endpoint (Fig. 2) — was significantly greater with DFN-11 than placebo (51.0% vs 30.8%, *P*  =  0.0023); the odds ratio (95% CI) was 2.57 (1.4–4.9). DFN-11 was also significantly superior to placebo for pain freedom at 30 min (22.3% vs 9.9%, *P*  =  0.0126), 60 min (34.6% vs 19.8%, *P*  =  0.0128), and 90 min postdose (41.3% vs 26.7%, *P*  =  0.0195), as shown in Fig. 2. Compared with placebo, a numerically greater proportion of subjects treated with DFN-11 reported absence of the MBS at 2 h postdose (64.1% vs 48.1%). A post hoc analysis using observed cases instead of LOCF imputation yielded the result that the difference between the proportion of those treated with DFN-11 and placebo for absence of the MBS was statistically significant (65.3% vs 47.4%, *P*  =  0.0210).

**Fig. 3:**
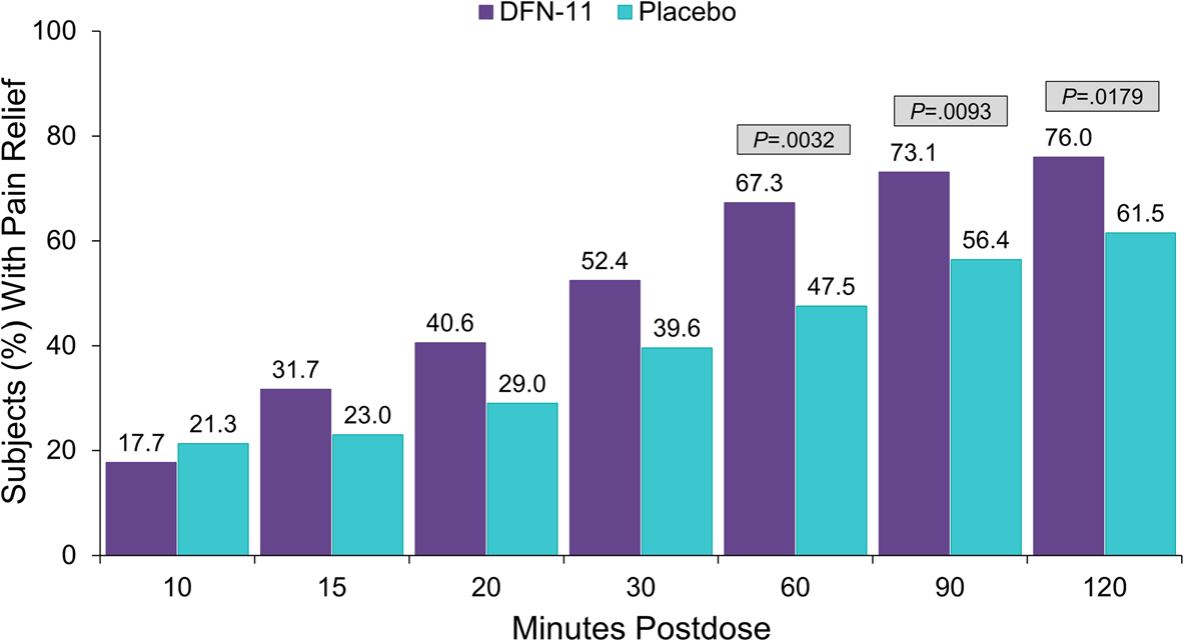
Proportion of subjects with pain relief through 2 h postdose

For pain relief (Fig. 3), DFN-11 was numerically superior to placebo beginning at 15 min postdose, and the differences between treatments were significant at 60 min (67.3% vs 47.5%, *P*  =  0.0032); 90 min (73.1% vs 56.4%, *P*  =  0.0093); and 2 h postdose (76.0% vs 61.5%, *P*  =  0.0179).

**Fig. 4:**
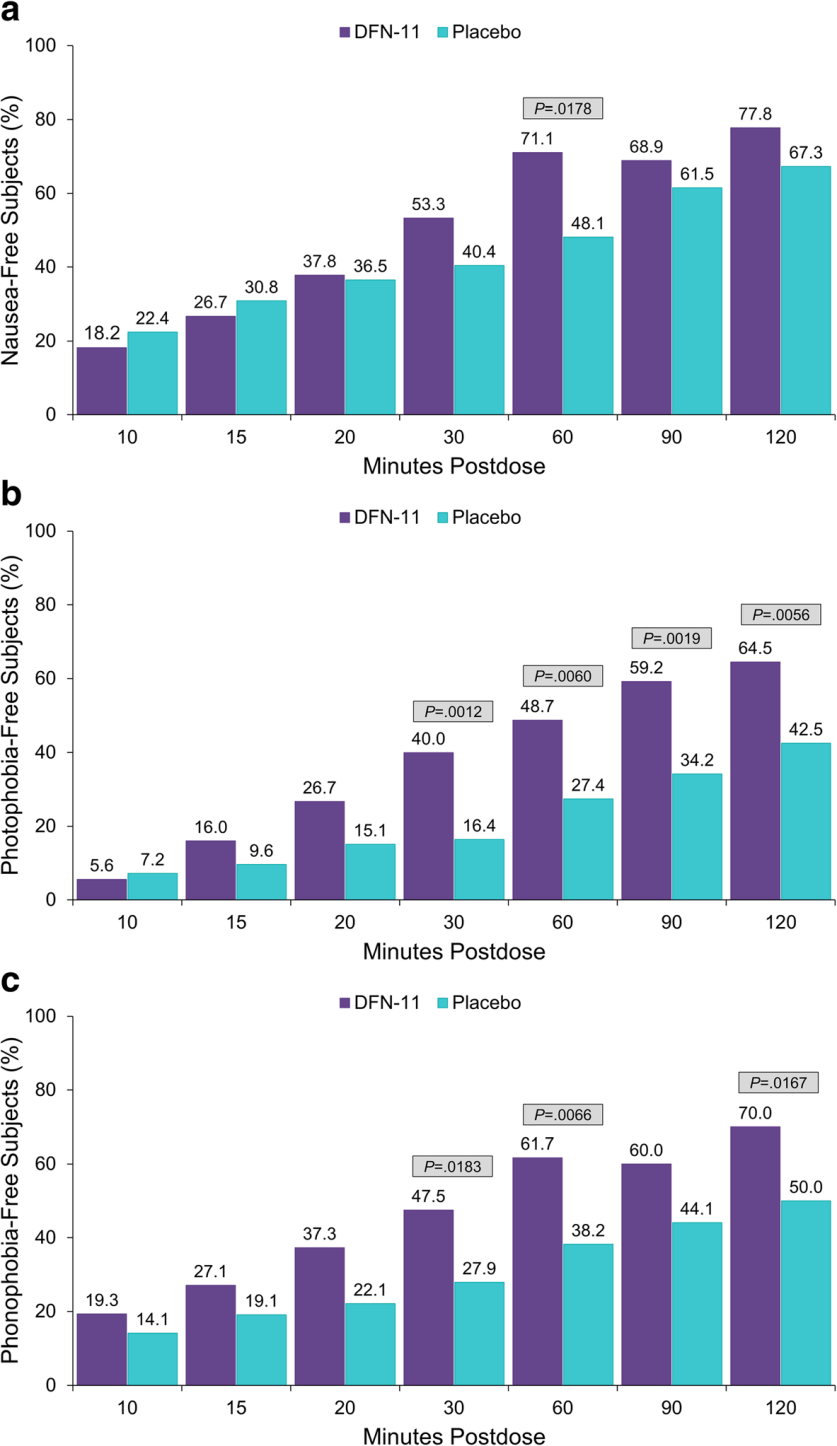
Proportions of subjects with freedom from nausea (**a**), photophobia (**b**), and phonophobia (**c**)

As shown in Fig. 4, DFN-11 outperformed placebo for freedom from nausea from 20 min postdose through 2 h postdose, and the separation was significant at 60 min postdose (71.1% vs 48.1%, *P*  =  0.0178). Compared with placebo, more subjects who were treated with DFN-11 were photophobia-free from 15 min through 2 h postdose, and the differences between DFN-11 and placebo were significant at 30 min (40.0% vs 16.4%, *P*  =  0.0012); 60 min (48.7% vs 27.4%, *P*  =  0.0060); 90 min (59.2% vs 34.2%, *P*  =  0.0019); and 2 h postdose (64.5% vs 42.5%, *P*  =  0.0056). Subjects in the DFN-11 group were more likely than those in the placebo group to be phonophobia-free from 10 min through 2 h postdose, and the separations from placebo were significant at 30 min (47.5% vs 27.9%, *P* = 0.0183); 60 min (61.7% vs 38.2%, *P* = 0.0066); and 2 h postdose (70.0% vs 50.0%, *P*  =  0.0167).

For sustained pain freedom from 2 through 24 h postdose, DFN-11 was numerically superior to placebo (74.4% vs 66.7%), and a smaller proportion of subjects took a second dose of study medication or rescue medication (25.2% vs 32.8%). On these endpoints, the differences between DFN-11 and placebo did not reach statistical significance.

Findings from the open-label portion of the study are beyond the scope of this manuscript and are reported elsewhere [23].

### Safety

During the double-blind treatment period, 23.0% of subjects (53/230) experienced at least 1 TEAE: 33.3% (37/111) of subjects who received DFN-11 and 13.4% (16/119) of those treated with placebo (Table 2). Most of these subjects (88.7%, 47/53) had events that were mild or moderate in intensity. Two subjects (1.8%) in the DFN-11 group had severe AEs (1 had injection site pain, and 1 had nausea). No subjects in the placebo group experienced severe AEs. Four subjects, 2 in each treatment group, had AEs of unknown severity. There were no serious AEs, but 2 subjects (0.9%) in the DFN-11 group had AEs that led to study discontinuation — worsening of migraine (not treatment-emergent) and a subject with 2 mild TEAEs of abnormal ECG (ventricular extrasystoles and sinus tachycardia), both of which were considered to be unrelated to the study drug.

**Table 2:** Treatment-emergent adverse events overall and occurring in at least 2 subjects

	DFN-11 (*n* = 111) *n* (%)	Placebo (*n* = 119) *n* (%)	Total (*N* = 230) *n* (%)
Subjects with ≥1 TEAE	37 (33.3)	16 (13.4)	53 (23.0)
Subjects with ≥1 injection site reaction	26 (23.4)	14 (11.8)	40 (17.4)
Injection site pain	8 (7.2)	7 (5.9)	15 (6.5)
Injection site swelling	8 (7.2)	1 (0.8)	9 (3.9)
Injection site bruising	5 (4.5)	3 (2.5)	8 (3.5)
Injection site irritation	4 (3.6)	3 (2.5)	7 (3.0)
Injection site erythema	2 (1.8)	1 (0.8)	3 (1.3)
Injection site induration	2 (1.8)	0 (0.0)	2 (0.9)
Paresthesia	3 (2.7)	0 (0.0)	3 (1.3)
Nausea	2 (1.8)	0 (0.0)	2 (0.9)
Throat tightness	2 (1.8)	0 (0.0)	2 (0.9)
Chest discomfort	1 (0.9)	2 (1.7)	3 (1.3)

*TEAE*, treatment-emergent adverse event

#### Injection site reactions

Table 2 shows that the most common TEAEs were associated with the injection site. In total, 17.4% (40/230) of subjects reported at least 1 injection site reaction, with 23.4% (26/111) in the DFN-11 group and 11.8% (14/119) in the placebo group. Compared with placebo, the rate among subjects who received DFN-11 was notably higher for injection site swelling (7.2% vs 0.8%).

#### Triptan-related adverse events

Triptan-related AEs (Table 3) were reported by 4.3% of subjects overall. They were generally more common among those treated with DFN-11 than with placebo (7.2% vs 1.7%), except for chest discomfort, which was less common in the DFN-11 treatment group (0.9% vs 1.7%), and dyspnea, reported by 1 subject (0.8%) in the placebo group and no subject treated with DFN-11.

**Table 3:** Triptan-related adverse events

	DFN-11 (*n* = 111) *n* (%)	Placebo (*n* = 119) *n* (%)	Total (*N* = 230) *n* (%)
Subjects with ≥1 triptan-related AE	8 (7.2)	3 (2.5)	11 (4.8)
Palpitations	0 (0.0)	1 (0.8)	1 (0.4)
Nausea	2 (1.8)	0 (0.0)	2 (0.9)
Chest discomfort	1 (0.9)	2 (1.7)	3 (1.3)
Dizziness	1 (0.9)	0 (0.0)	1 (0.4)
Lethargy	1 (0.9)	0 (0.0)	1 (0.4)
Paresthesia	3 (2.7)	0 (0.0)	3 (1.3)
Dyspnea	0 (0.0)	1 (0.8)	1 (0.4)
Throat irritation	1 (0.9)	0 (0.0)	1 (0.4)
Throat tightness	2 (1.8)	0 (0.0)	2 (0.9)

*AE*, adverse event

## Discussion

DFN-11 was significantly more effective than placebo for the primary endpoint, the proportion of pain free subjects at 2 h postdose, which is currently a guidelines-recommended primary measure of efficacy in migraine trials [25]. Significant separation from placebo was seen starting at 30 min postdose, meeting both patient need for rapid migraine relief [26, 27] and guideline recommendations for measurements of pain freedom before 2 h postdose [25]. DFN-11 was also significantly better than placebo for migraine pain relief from 60 min through 2 h postdose. In addition to significant superiority over placebo on pain free and pain relief outcomes, DFN-11 provided significantly better relief of the symptoms associated with migraine, including the subject-identified MBS (in post hoc analysis). These positive responses on multiple efficacy endpoints suggest that DFN-11 has attributes that are clinically important to migraine sufferers, specifically, fast and effective relief of migraine pain and associated symptoms [26, 27].

As expected, the most common TEAEs were related to the injection site, with a higher overall incidence for DFN-11 than placebo. It is noteworthy, however, that injection site reactions following DFN-11 in the current study were 61% lower than those previously reported for SC sumatriptan (23% vs 59%) [17]. Similarly, the incidence of triptan sensations with the 3 mg dose of sumatriptan in DFN-11 was low. Although not directly comparable, the percentage of subjects with triptan sensations associated with DFN-11 in this study was approximately 80% lower than published estimates for the 6 mg SC dose (7.2% vs 42%), and DFN-11 demonstrated lower rates than placebo on chest discomfort. Treatment-emergent AEs of dyspnea and palpitations were only experienced in the placebo group.

Administration of sumatriptan via SC injection is one option for patients in whom oral medications are suboptimal, but needle-averse patients seeking a more rapid onset of action than oral agents can provide may benefit from intranasal sumatriptan [28]. A novel intranasal powder form is available (ONZETRA® Xsail®, AVANIR Pharmaceuticals, Aliso Viejo, CA, USA), and an intranasal spray (sumatriptan 10 mg with a permeation-enhancing excipient) remains in development, and both of these agents have a fast onset of action and are effective in the acute treatment of migraine [29–31]. For patients in whom speed of onset is the most important attribute of an acute migraine medication, however, the SC injection of sumatriptan remains the fastest and most effective therapy.

The results of this study support the efficacy and safety of the 3 mg autoinjector dose of sumatriptan, and they complement the results of a previous randomized, double-blind, crossover trial with DFN-11 [22], which compared the 3 mg dose with a 6 mg dose of DFN-11 [22]. In this pilot trial, a single 3 mg dose of DFN-11 provided relief of migraine pain and associated symptoms comparable to 6 mg and was associated with fewer triptan sensations. Achieving similar efficacy and safety results compared with placebo in the current well-powered, double-blind study supports that patient response to DFN-11 is likely to be reliable and predictable.

This study met its primary efficacy endpoint and demonstrated the efficacy and tolerability of DFN-11. These results, which provide the first placebo-controlled data on the 3 mg sumatriptan SC autoinjector for the acute treatment of migraine, should be useful in predicting response to DFN-11 in clinical practice. Limitations of the study include the high placebo response and the inability of a single-attack, parallel-group design to detect fluctuations in treatment response across multiple attacks. Future studies, are needed to further clarify the clinical utility and therapeutic potential of DFN-11.

## Conclusions

In this multicenter, randomized, double-blind, placebo-controlled study in adults with episodic migraine, DFN-11 was significantly more effective than placebo on multiple pain free and pain relief assessments from 30 min through 2 h postdose and the relief of migraine associated symptoms, including the subjects’ MBS. Taken together with the low incidence of TEAEs and triptan sensations, these findings demonstrate that the 3 mg dose of sumatriptan in DFN-11 may be a useful alternative to the 6 mg SC dose of sumatriptan.

## Additional file

Additional file 1: Exclusion criteria. (DOCX 39 kb)

## Abbreviations

AE: Adverse event
ATC: Anatomic Therapeutic Chemical
ECG: Electrocardiogram
eDiary: Electronic diary
ICHD: International Classification of Headache Disorders
IRB: Institutional Review Board
LOCF: Last observation carried forward
MBS: Most bothersome symptom
MedDRA: Medical Dictionary for Regulatory Activities
NSAID: Nonsteroidal anti-inflammatory drug
SAE: Serious adverse event
SC: Subcutaneous
SD: Standard deviation
TEAE: Treatment-emergent adverse event
WHODDE: World Health Organization Drug Dictionary Enhanced
