# Supplementary material for: Efficacy and safety of DFN-11 (sumatriptan injection, 3 mg) in adults with episodic migraine: a multicenter, randomized, double-blind, placebo-controlled study
Source: J Headache Pain. 2018 Aug 15;19(1):69. doi: 10.1186/s10194-018-0881-z (PMC6093827; doi:10.1186/s10194-018-0881-z)
Supplement: Supplementary file 1 — Exclusion criteria. (DOCX 39 kb) [file 10194_2018_881_MOESM1_ESM.docx]

# Appendix

## Exclusion criteria

Subjects were excluded for any of the following reasons: medication overuse headache, as defined by the Second Edition of International Classification of Headache Disorders (ICHD-2) [1]; use of opioids, other pain medications, combination analgesics, triptans, or ergot alkaloids or any combination of these medications 10 or more days per month during the 90 days prior to screening; NSAIDs or simple analgesics on more than 14 days per month during the 90 days prior to screening; botulinum toxin treatment within 180 days before screening; unstable dosages of migraine preventive medications during the 30 days prior to and through screening; mini-prophylaxis for menstrual migraine. Subjects were also excluded if they had hemiplegic or basilar migraine or other forms of neurologically complicated migraine; cerebrovascular disease; a history of migralepsy; inability to differentiate between migraine and other types of headache; a history of more than occasional tension-type headache (distinct from migraine headache days); cluster headaches; prior diagnosis of ICHD-2 probable migraine; ischemic coronary artery disease; Wolff-Parkinson-White syndrome or arrhythmias associated with other cardiac accessory conduction pathway disorders; a history of congenital heart disease; uncontrolled hypertension on screening; peripheral vascular disease; or any abnormal physiology and/or pathology that would be contraindicated for study participation and would not allow the objectives of the study to be met.

Additional reasons for exclusion from study participation included any clinical laboratory or electrocardiogram (ECG) abnormality that would endanger subjects or interfere with the study conduct; Fridericia's corrected QT interval greater than 450 msec; severe renal impairment (creatinine >2 mg/dl); serum total bilirubin >2.0 mg/dL; serum aspartate aminotransferase, alanine aminotransferase, or alkaline phosphatase greater than 2.5 times the upper limit of normal; uncontrolled diabetes mellitus; glycosylated hemoglobin (HbA1c) >7.0%; diabetes mellitus requiring insulin; history of alcohol or substance use disorder (including marijuana) within 1 year prior to screening; current treatment with antipsychotics or use of antipsychotics within 30 days of screening; history of or current neurological or psychiatric impairment (eg, psychosis, current major depression, bipolar disorder, or cognitive dysfunction) that would compromise data collection; treatment with an investigational drug or device within 30 days of the screening visit; participation in a central nervous system clinical trial in the 3 months prior to screening; any other medical condition (eg, positive screening test for human immunodeficiency virus [HIV], hepatitis B surface antigen positive or hepatitis C positive, a known history of systemic lupus erythematosus) that would confound the objectives of the study; a plan to donate blood, sperm, or oocytes during the study and for 30 days after the last dose of study medication; status as an employee or immediate relative of an employee of the Sponsor, any of its affiliates or partners, or of the study centers.

**Reference**

1. The International Classification of Headache Disorders: 2nd edition (2004). Cephalalgia 24 Suppl 1:9-160
